# Physical activity and dietary habits of older children and adolescents in Germany – Cross-sectional results of the 2017/18 HBSC study and trends

**DOI:** 10.25646/6900

**Published:** 2020-09-16

**Authors:** Jens Bucksch, Angela Häußler, Katja Schneider, Emily Finne, Katrin Schmidt, Kevin Dadacynski, Gorden Sudeck

**Affiliations:** 1 University of Education Heidelberg Faculty of Natural and Sociological Sciences, Department of Prevention and Health Promotion; 2 University of Education Heidelberg Heidelberg Centre for Prevention and Health Promotion; 3 University of Education Heidelberg Faculty of Natural and Sociological Sciences, Department of Nutrition and Home Economics; 4 Bielefeld University School of Public Health, Department Prevention and Health Promotion; 5 Eberhard Karls University Tübingen Institute of Sport Science; 6 Fulda University of Applied Sciences Department of Nursing and Health Science, Fulda Public Health Centre; 7 Eberhard Karls University Tübingen Interfaculty Research Institute for Sport and Physical Activity

**Keywords:** PHYSICAL ACTIVITY, DIET, TRENDS OVER TIME, PREVALENCE, HBSC, HEALTH REPORTING

## Abstract

Numerous findings are known to exist between dietary habits, physical activity, and child and adolescent health. Here, we will use data from the most recent Health Behaviour in School-aged Children (HBSC) study to describe dietary habits and patterns of physical activity. Using the survey data for 11-, 13- and 15-year-old students from across Germany, we report findings for key indicators of diet and physical activity for the 2017/18 cycle. By comparing these findings with data from the 2009/10 and 2013/14 survey cycles, we also consider current trends. Results from the most recent cycle show that 10.0% of girls and 16.9% of boys meet the World Health Organization’s (WHO) physical activity recommendations. Across all HBSC cycles, this is the lowest figure so far. Concerning dietary habits, 50.6% of girls and 59.0% of boys reported having breakfast every morning. Data for daily fruit, vegetable and soft drink consumption emphasises the need to promote a healthy diet among adolescents. For all indicators of physical activity and diet, differences between girls and boys are apparent. Girls’ intake of fruit and vegetables is higher and they consume fewer soft drinks, yet boys are more physically active and have breakfast more regularly. For the majority of indicators of dietary habits and physical activity, considerable inequalities relating to family affluence are observed. An important implication of the study results for dietary habits and physical activity of older children and adolescents is the need to foster settings-based approaches to promote physical activity and a healthy diet that integrate a gender-sensitive perspective.

## 1. Introduction

Sufficient physical activity and a balanced diet are important factors that influence a person’s health over their lifetime. Already during childhood and adolescence, high levels of physical activity provide benefits to physical and mental health [1]. In a similar manner, eating fruit and vegetables every day, regularly having breakfast daily, as well avoiding the regular consumption of soft drinks, benefit a person’s physical and cognitive development [2]. The positive effects of physical activity and diet during childhood and adolescence on the healthy development of body weight, bone health and higher academic achievement illustrate this fact [3–6].

In combination, physical activity and diet play an important role in balancing energy uptake and expenditure [1, 4]. Moreover, as ample evidence exists for the effects of physical activity and dietary habits on the development of chronic disease at adult age [7, 8], this highlights the need to provide initiatives for and to promote health-benefiting levels of physical activity and healthy dietary habits among children and adolescents [9, 10].

During recent years, diverse recommendations for physical activity have been developed. Among them are Germany’s recommendations for physical activity behaviour and the promotion of physical activity, which were published on behalf of Germany’s Federal Ministry of Health (BMG) and recommend a minimum target of 90 minutes of physical activity of at least moderate intensity per day for children and adolescents [11]. Moderate intensity physical activities include, for example, brisk walks or other physical activities that lead to a slight increase in heart rate and breathing, but where talking is still possible. Vigorous intensity activities accelerate a person’s heart rate further, cause them to sweat and become out of breath, therefore making it difficult to talk to others. Health-oriented physical activity recommendations for children and adolescents usually refer to this differentiation of exercise intensity, and include at least moderate intensity activities during daily life and leisure time. Doing sports is a specific form of physical activity usually related to a particular type of sport or structured training. Sports can make up an important part of vigorous physical activity and would then also contribute to health-enhancing moderate to vigorous physical activity (MVPA). The World Health Organization’s (WHO) recommendations on physical activity already consider a daily minimum of 60 minutes of moderate intensity physical activity as providing children and adolescents with substantial health benefits. Compared to Germany’s national recommendations, it is therefore considered that already these lower levels provide health benefits [12].

In spite of the obvious health benefits linked to dietary habits and levels of physical activity, from a population perspective, the question remains how many children and adolescents actually behave in line with these recommendations. According to an international comparative study, at a global level, 15.3% of 11- to 17-year-old girls and 22.4% of boys in the same age range met the WHO recommendations for physical activity. Figures for Germany (20.3% and 12.1%, respectively) would then be below the global average [13]. If we more narrowly focus on sports, further studies indicate that many children and adolescents regularly engage in sports. However, it remains rather unclear how far sport contributes to older children and adolescents fulfilling the physical activity recommendations. For sports, considerable differences between the sexes and regarding socioeconomic status were repeatedly observed to the detriment of girls and socially marginalised groups. These differences are also visible, although are less pronounced, in the figures for those who meet the WHO physical activity recommendations for total health-benefiting physical activity [14, 15].

The recommendations on dietary habits include those augmented by the German Nutrition Society (DGE) [16], as well as by the WHO [17]. Three portions of vegetables and two portions of fruit daily are recommended [16], as well as sufficient energy-free or low-energy soft drinks to reduce the consumption of free sugars to less than 10% of daily energy uptake [17]. Data from the second follow-up to the German Health Interview and Examination Survey for Children and Adolescents (KiGGS Wave 2) found that only one in six children met the recommended ‘five portions daily’ of fruit and vegetables [23]. Earlier HBSC study data also showed potential for improvement in dietary habits. By sex, girls do better [14, 15].

Such findings from health reporting have already led to initiatives to align and strengthen a number of measures to promote physical activity and healthier dietary habits, for example the National Action Plan ‘In Form – German national initiative to promote healthy diets and physical activity’ [18]. Against this backdrop, and based on recent data, this paper presents findings from the 2017/18 Health Behaviour in School-aged Children (HBSC) study on physical activity and dietary habits during late childhood and adolescence. Moreover, trends are described that provide us with a wider picture of the analysed survey variables across the three last survey cycles.

## 2. Methodology

### 2.1 Sample design and study implementation

The analyses in this paper are based on German HBSC study data. Mostly we rely on data that was collected in the 2017/18 school year, with a total of 2,306 girls and 2,041 boys in the three age groups of 11-, 13-, and 15-year-olds surveyed. To analyse trends for our indicators, we also used data from previous survey cycles that used identical surveying methods in Germany-wide samples during 2009/10 [14] and 2013/14 [15]. For the study variables covered here, neither answers nor answering formats changed over time. During all surveying points, HBSC relied on a standardised protocol that is assembled within the International Coordinating Centre for each survey cycle. The survey instruments have been successfully used for years and the reliability as well as the validity of the indicators used here have been examined by numerous studies with satisfactory or good results [19]. Moor et al. in this issue of the Journal of Health Monitoring provide a detailed description of the study design and background.

### 2.2 Survey instruments

The focus of this paper are indicators of physical activity and dietary habits, with sex, age and family affluence used as socioeconomic stratification variables. Sex is determined through the dichotomous answer of ‘girl’ or ‘boy’. Age is defined through the age categories 11, 13 or 15 years. To measure the social status of adolescents, HBSC surveys socioeconomic status based on family affluence (FAS). A detailed description is included in Moor et al. in this issue of the Journal of Health Monitoring.

#### Indicators of physical activity and sports

Physical activity of at least moderate intensity was measured by asking how often during the last seven days the older children and adolescents had been physically active for at least 60 minutes. Examples were provided to explain that this includes any kind of physical activity during the day that increases pulse rate and leaves you out of breath for some time. Participants could tick one out of eight answer boxes between zero and seven days. With these answers, two indicators were then created. First, it was determined whether students had been moderately physically active during at least 60 minutes per day and had therefore engaged in levels of physical activity that are considered to promote health (‘WHO physical activity recommendations fulfilled’). Secondly, those students who answered between zero and two days, i.e. that had been moderately physically active for 60 minutes during less than three days per week, were identified (‘low levels of physical activity’).

In addition to information on general physical activity, the HBSC study also collects data on sports. As the answers for our first indicator (sometimes called the MVPA Indicator, i.e. Moderate-to-Vigorous Physical Activity) refer to any kind of activity during leisure time and daily life, the response actually includes sports-related activities solely/only. For a more specific analysis of sports with its often higher intensity and particular forms of organisation (such as sports clubs and fitness studios), participants were asked how often they engaged in sports activities during leisure time with an intensity that left them out of breath and/or sweating. Students were able to choose from seven answers ranging from ‘every day’ to ‘never’. The answers were dichotomised for analysis in accordance with international reports such as the HBSC study [19], and used sports activities during at least four days per week as the reference value.

#### Diet-related indicators

Dietary habits are described based on fruit, vegetable and soft drink (defined as Cola or other sugary lemonades) consumption. The survey asked how often these had been consumed on a seven-point scale ranging from ‘never’ to ‘several times per day, every day’. Based on the current DGE and WHO recommendations, the analyses present the proportion of participants who eat fruit and vegetables at least daily and drink soft drinks at least less than daily. A further indicator was the number of school days on which girls and boys ate breakfast at home before going to school. Breakfast was defined as ‘more than a glass of milk or juice’. The analyses refer to the proportion of participants that have breakfast every day versus those who do not. The indicator shows the frequency with which participants have breakfast as a health-relevant dimension [3, 6]. All indicators of physical activity and diet were operationalised to ensure their comparability with international HBSC reporting standards [19].

### 2.3 Statistical methods

The core results are presented descriptively as prevalences or percentage frequencies with 95% confidence intervals (CI) and separately for girls and boys. The results are also stratified by age and family affluence. Discrepancies in the total number of girls and boys in the tables are owed to missing data for individual variables. To statistically secure this descriptive information regarding the sample subgroups, binary logistic regressions were calculated. Correlations between the sociodemographic markers of sex, age and family affluence and behaviour-related variables were estimated based on regression models that adjusted all of the variables. The results are presented as odds ratios (OR) and 95% CI. Trends are described for all the variables relevant to diet and physical activity for the last three survey cycles through percentage frequencies for both sexes.

All analyses were conducted with SPSS 24. To optimise representativeness, a weighting factor was introduced. The weighting factors correct for slight differences between proportions by federal state and type of school in the achieved sample with regard to the proportions previously defined based on school statistics. A detailed description is included in Moor et al. in this issue of the Journal of Health Monitoring. Absolute figures in the tables refer to the unweighted data. Regression model prevalences and effects estimators are weighted in the report.

## 3. Results

###  

#### Indicators of physical and sports-related activities

10.0% of girls and 16.9% of boys fulfilled the WHO recommendations for physical activity. The proportion of girls and boys that fulfil the WHO physical activity recommendations decreases with age. Logistic regression analyses highlight statistically significant differences for sex and age groups. 15-year-old girls thereby achieved the lowest values, with only 7.3% stating that they were physically active every day for at least 60 minutes. With regard to family affluence, the highest values for girls (14.5%) and boys (22.4%) were found for affluent families; however, this was not statistically significant in the multivariate model (Table 1 and Table 2).

One fifth of boys and one third of girls were physically active for 60 minutes on less than three days per week, and therefore categorised as having low levels of physical activity. This difference by sex is statistically significant. By age, low levels of physical activity were more pronounced among 15-year-old girls and boys compared to the two younger age groups, with no significant statistical differences found between 11- and 13-year-old girls and boys (Table 2). Furthermore, there was an inverse relationship between the proportion of girls and boys in the low physical activity group and family affluence: whereas two out of ten girls with high family affluence were physically less active, four out of ten girls in the low family affluence group were less physically active per week.

For sports, similar patterns emerged: in particular boys, younger students and girls and boys from high affluence families considerably more frequently participated in sports-related activities on more than four days per week. Around half of all boys fulfilled this criterion, yet only about one in three girls performed sports activities at least four times per week. Moreover, levels of sport decreased with age.

**Figure 1:**
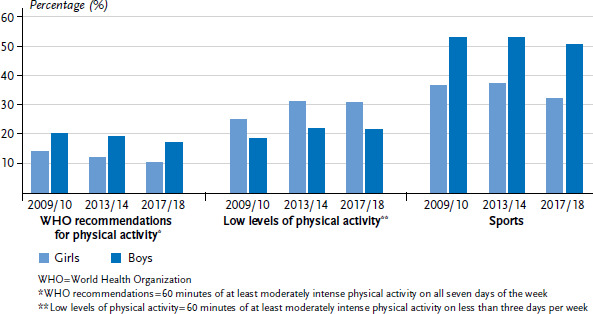
Comparison of indicators ‘WHO recommendations for physical activity’, ‘low levels of physical activity’ and ‘sports (at least four times per week)’ by sex across the HBSC survey cycles 2009/10 (n=2,525 girls, n=2,364 boys), 2013/14 (n=2,857 girls, n=2,967 boys), 2017/18 (n=2,278 girls, n=2,021 boys) Source: 2009/10, 2013/14 and 2017/18 German HBSC study

Figure 1 shows prevalence trends for the three indicators of physical activity over the last three HBSC survey cycles. Numbers for fulfilling the WHO physical activity recommendations decreased for both sexes (for girls from 14.0% to 10.1% and for boys from 20.0% to 16.9%). The low level of physical activity increased from 2009/10 to 2013/14 by six percentage points for girls (from 24.8 % to 30.6 %) and about three percentage points for boys (from 18.5% to 21.3%). For both girls and boys, the values for 2017/18 have remained largely stable since the previous cycle 2013/14. In line with these findings, compared to previous cycles, the 2017/18 cycle observed the lowest numbers for older children and adolescents that engage in sports activities at least four times per week. This decrease is particularly marked for girls between 2013/14 and 2017/18 (from 37.1% to 31.8%).

#### Dietary indicators

50.6% of girls and 59.0% of boys have breakfast at home daily (Table 3). The regression model found the differences between girls and boys, as well as the decrease in figures for regular breakfast with age and lower family affluence, to be statistically significant. In the high family affluence group, for example, considerably more girls and boys had breakfast compared to the lower status group, in which merely 39.0% and 46.2% of girls and boys respectively reported having breakfast every day (Table 2).

42.1% of girls and 32.1% of boys ate fruit every day. Figures for daily vegetable intake were lower for both sexes (Table 3). Logistic regression analysis showed the differences by sex, a decrease in intake levels with age, as well as the increase in fruit intake from high to low family affluence to be statistically significant (Table 2).

88.5% of girls and 82.5% of boys do not consume soft drinks every day. The correlation between frequent soft drink consumption, age and lower family affluence was found to be statistically significant. In all age groups, girls consumed soft drinks daily less frequently than boys (Table 3). Multivariate regression models showed this to be statistically significant (Table 2).

**Figure 2:**
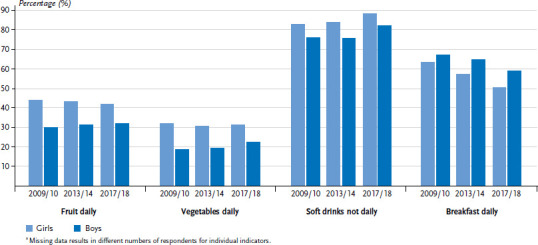
Comparison of daily fruit and vegetable intake, non-daily soft drink consumption and daily breakfasting by sex for HBSC survey cycles 2009/10 (n=2,563 girls, n=2,416 boys), 2013/14 (n=2,908 girls, n=3,003 boys), 2017/18 (n = 2,280 girls, n = 2,002 boys)* Source: 2009/2010, 2013/2014 and 2017/2018 German HBSC study

Figure 2 shows the trend for prevalences of the four indicators of dietary habits. Across the survey cycles, the number of girls and boys that had breakfast daily has decreased continuously (from 63.6% to 50.6% and 67.3% to 59.0% for girls and boys, respectively). For fruit and vegetable intake, the picture was mixed. During all three survey points, more girls than boys ate fruit and vegetables every day. However, daily fruit and vegetable consumption slightly increased for boys during the survey period (by 2.1 percentage points for fruit and 3.7 percentage points for vegetables), while for girls a slight decrease was observed (by 1.9 percentage points for fruit and 0.6 percentage points for vegetables). In particular between survey cycles 2013/14 and 2017/18, the figures for not drinking soft drinks every day increased for both sexes (from 84.0% to 88.5% and 76.0% to 82.5% for girls and boys, respectively).

## 4. Discussion

The HBSC data from the 2017/18 survey cycle was described through a set of indicators based on self reported data on physical activity and dietary habits. Generally, the data shows a need for actions to promote physical activity and a healthy diet during childhood and adolescence.

###  

#### Indicators of physical activity and sports

In light of the finding that only one in ten girls, and one in six boys, fulfill the WHO recommendations, we must point out a widespread lack of physical activity in the 11-, 13- and 15-year-old age group. This situation has worsened over time. Further studies corroborate our findings and, compared to other high-income western countries, the figures for Germany are below average [13, 20–22]. In addition, KiGGS Wave 2 data shows that figures for meeting the recommended levels of physical activity have decreased between Wave 1 (2009–2012) and Wave 2 (2014–2017), in particular for 3- to 10-year-old girls. For all other groups of children and adolescents, the figures are stable but remain at a low level [22].

Remarkably, our data shows that the proportion of girls and boys that have low levels of physical activity has increased over time. This means that, in addition to the slight decrease in those who engage in enough physical activity to have health benefits, there has been a substantial increase in the number of older children and adolescents whose levels of physical activity are clearly too low. This observation is in line with KiGGS study results [22] highlighting the need for a differentiated analysis specifically of highly sedentary girls and boys. Importantly, our survey instrument thereby does not allow respondents to be differentiated according to the degree by which they fulfil the recommended 60 minutes of daily physical activity. From a health perspective, this lack of precision is relevant too, as it does make a difference whether the recommended levels of activity were missed by 5 or by 50 minutes.

For the frequency of sports, which is a specific aspect of overall physical activity, current HBSC study data confirms findings of a relatively high prevalence, which is skewed towards boys [15, 21]. Presumably, therefore, sport is a factor that, overall, raises figures for fulfilling the recommended healthy levels of physical activity among boys. However, with regard to reaching levels of physical activity beneficial to health, sports represent the most important physical activity for a relatively small proportion of adolescents [23]. Comprehensively promoting physical activity among older children and adolescents will therefore require a combined strategy that not only promotes participation in structured sports, but also generally promotes physical activity in leisure time and daily life.

#### Indicators of dietary habits

The figures for daily soft drink consumption have decreased and levels of fruit and vegetable intake remain stable at levels with a potential for improvement. Yet with regard to having breakfast, our data points to a negative development. This finding is in line with other (inter)national findings [14, 15, 24–26].

A closer analysis of individual indicators shows some issues worthy of discussion. The decrease in the frequency with which older children eat breakfast every morning is at least partially due to age-typical developments. Daily and sleep rhythms change during adolescence, and breakfast time often competes with sleeping time [27]. Furthermore, distancing oneself from one’s parents is normal at this age and can lead adolescents to no longer regularly take part in family meals [28]. An increasing orientation towards body ideals and more frequent dieting explain the particularly low prevalence found for older girls [29]. A potential weakness of this indicator is that students could hypothetically also regularly have breakfast at school, a fact not reflected by the corresponding question. Broadening the question in this manner, however, would not reflect school realities. Schools do not offer breakfast before lessons, and having breakfast later, for example during the first longer break, has drawbacks, such as with regard to body energy requirements.

The results for fruit and vegetable intake reveal a noticeably clear trend, showing that children and adolescents are not meeting the DGE recommendations [16]. One weakness worth taking into account is that the applied measurement instrument does not entirely reflect the recommendations. KiGGS Wave 2 data corroborates our results [26]. Surveys of the adult population also indicate low levels of vegetable intake in particular [30]. Intervening early in the school environment would therefore promising, for example by promoting a higher fruit and vegetable intake through novel taste experiences.

With age, the number of students who say that they consume soft drinks daily decreases. KiGGS data is in line with this primarily positive finding and confirms that sugary drinks consumption decreases across all groups, with a concurring considerable rise in the amount of water consumed [26]. International data, too, supports this positive trend [25]. It cannot be ruled out, however, that greater public awareness concerning sugary drinks may have distorted the responses. However, it is just as possible that the installation of, for example, water dispensers in schools and the provision of drinks in classrooms offer a realistic explanation for this positive development.

#### Broader context

For all indicators of dietary habits and physical activity, considerable differences between girls and boys were evident. While on average a majority of older children and adolescents do not fulfill the recommendations for diet, the dietary habits of girls, as much regarding fruit and vegetable intake as soft drink consumption, are clearly healthier across all surveys [29]. The opposite is true for physical activity. HBSC data confirms the findings of other studies such as KiGGS [22, 26]. As has been observed, the importance of gender-specific behaviour as an expression of gender identity increases in particular during adolescence. While female adolescents are more oriented towards slim body ideals and increasingly diet to lose weight (for example by not having breakfast), male adolescents are driven by athletic body ideals and the desire to develop a muscular physique [31]. These gender ideals frequently appear, particularly in nutritional campaigns (e.g. through the use of body images), as a starting point for a supposedly target-group orientated approach to healthy eating. However, such approaches only serve to further reinforce gender stereotypes. New prevention measures should therefore aim not to further strengthen stereotypes, and must achieve this by reflecting on gender differences and using gender-sensitive forms of communication [32].

Further expanding systematic measures to promote physical activity and a healthy diet should therefore specifically take the gender perspective into account (in particular during and after puberty). Different factors create the conditions that lead girls and boys to change their behaviour, and these factors are, not least, context and behaviour specific (for example regarding active transport [33]). Moreover, findings from nutritional and sports science on the positive effects of behaviour indicators need to be considered and communicated to the target group, so that health competency can become the basis of decision-making. Though many may not be aware, a regular breakfast can, for example, benefit performance and concentration as well as positively influence weight regulation [24, 34, 35].

For the majority of indicators, 2017/18 HBSC data clearly shows that socioeconomic status continues to play a role in further reinforcing health inequalities. In spite of increasing awareness, results from various studies indicate that these inequalities have not decreased during recent years [25]. For some indicators, the differences are substantial (for example regarding participation in sports), whereby a lack of financial means or other barriers to participation are possible explanations. For diet and physical activity, the most recent HBSC data emphasises a need to make greater efforts for equal social opportunities, and the data should be used more intensively for future action in the fields of health promotion and prevention. Not least, this implies the need for an even greater focus on settings-based perspectives to promote physical activity and a healthy diet. For example, efforts should be increased to intensify approaches that aim to structurally anchor the availability of attractive and healthy food options for snacks and lunch at school. Given the generally positive effects on performance and concentration, offering balanced breakfasts at school could create more equality in education and of opportunities, in particular regarding children and adolescents who come to school without having eaten breakfast [36]. Taking into account adolescent eating habits [28], schools could contribute by offering innovative snacks with a relevant proportion of fruit and vegetables [37].

The chances of success for a targeted development of measures that are also settings-based appears to depend, on the one hand, on more actively integrating socially disadvantaged groups into the design and implementation of measures [36–38]. On the other hand, focusing solely on the school setting is not enough and schools must be considered within a wider socioecological context. In the promotion of physical activity, there appear to be some positive examples [39, 40]. However, we need to recognise that overall there is too little knowledge on why and under which circumstances a particular intervention is effective in various target groups of children and adolescents [41, 42].

#### Conclusion

For dietary habits and physical activity in general, a set of prevalence patterns depending on sex, age and family affluence can be distinguished, and it would be important to discuss the implications with regard to developing a better combination of behaviour and setting-based approaches [43]. While the empirical research on increasing equal opportunities in society through intervention measures during childhood and adolescence remains limited [42], findings indicate [33, 44] that strengthening settings-based approaches would increase the likelihood of successfully increasing levels of physical activity and healthier dietary habits among all children and adolescents.

## Key statements

- Across the survey cycles, fewer girls (2017/18: 10%) and boys (2017/18: 16.9%) meet the WHO physical activity recommendations.
- In recent years, a decreasing number of older children and adolescents have breakfast every morning, yet they also consume soft drinks less frequently.
- While boys are more physically active than girls, a majority of girls score better in indicators of diet.
- Fruit and vegetable consumption should be promoted among older children and adolescents in line with the recommendations made by the German Nutrition Society (DGE).
- Family affluence is closely connected to the dietary habits of older children and adolescents. In physical activity levels, this is true in particular for sports activities. For overall physical activity, the correlations with family affluence are less consistent.

## Figures and Tables

**Table 1:** Physical activity and sports by sex, age and family affluence (n=2,278 girls, n=2,021 boys) Source: 2017/18 German HBSC study

	WHO recommendations for physical activity^*^		Low levels of physical activity^**^		Sports (≥ 4 days per week)	
	%	(95 % CI)	%	(95 % CI)	%	(95 % CI)
**Girls**	**10.0**	**(8.8–11.4)**	**30.6**	**(28.6–32.6)**	**31.9**	**(29.9–34.0)**
**Age group**						
11 years	14.3	(11.8–17.2)	23.9	(20.7–27.4)	42.7	(38.9–46.6)
13 years	9.1	(7.1–11.6)	27.5	(24.2–31.0)	30.3	(26.8–33.9)
15 years	7.3	(5.6–9.3)	38.9	(35.6–42.4)	23.9	(21.1–27.1)
**Family affluence**						
Low	10.3	(7.7–13.6)	40.3	(35.5–45.2)	25.0	(21.0–29.5)
Medium	8.7	(7.2–10.4)	30.6	(28.1–33.1)	30.8	(28.3–33.4)
High	14.5	(11.2–18.6)	19.7	(15.9–24.1)	44.2	(39.2–49.4)
**Boys**	**16.9**	**(15.2–18.7)**	**21.3**	**(19.5–23.3)**	**50.2**	**(47.9–52.6)**
**Age group**						
11 years	21.2	(18.1–24.7)	19.0	(16.0–22.4)	58.7	(54.7–62.6)
13 years	16.4	(13.7–19.5)	19.7	(16.8–23.1)	51.3	(47.4–55.3)
15 years	12.9	(10.4–15.9)	25.4	(22.1–29.1)	40.0	(36.2–44.0)
**Family affluence**						
Low	19.0	(14.7–24.1)	31.2	(26.2–37.1)	45.9	(40.1–51.9)
Medium	15.1	(13.2–17.3)	20.4	(18.2–22.7)	48.6	(45.8–51.4)
High	22.4	(17.9–27.6)	14.9	(11.3–19.6)	62.4	(56.6–67.9)

CI = Confidence interval, WHO = World Health Organization

^*^ WHO recommendations = 60 minutes of at least moderately intense physical activity on all seven days of the week

^**^ Low levels of physical activity = 60 minutes of at least moderately intense physical activity on less than three days per week

**Table 2:** Odds ratios and 95% confidence intervals for indicators of physical activity and dietary habits by sex, age and family affluence (multivariate logistic regression model including all predictors) Source: 2017/18 German HBSC study

	WHO recommendations for physical activity^*^ (n=4,219)		Low levels of physical activity^**^ (n=4,219)		Sports (≥ 4 days per week) (n=4,197)	
	OR	(95 % CI)	OR	(95 % CI)	OR	(95 % CI)
**Age group**						
11 years	1.00		1.00		1.00	
13 years	0.68	(0.55–0.84)	1.11	(0.93–1.33)	0.68	(0.58–0.79)
15 years	0.53	(0.42–0.66)	1.68	(1.41–2.00)	0.46	(0.39–0.53)
**Family affluence**						
Low	1.00		1.00		1.00	
Medium	0.79	(0.63–1.01)	0.60	(0.51–0.71)	1.23	(1.04–1.47)
High	1.30	(0.98–1.73)	0.38	(0.29–0.48)	2.10	(1.69–2.62)
**Sex**						
Boys	1.00		1.00		1.00	
Girls	0.54	(0.45–0.65)	1.59	(1.38–1.83)	0.46	(0.41–0.53)

	Fruit daily (n=4,196)		Vegetables daily (n=4,195)		Soft drinks not daily (n=4,200)		Breakfast daily (n=4,200)	
	OR	(95 % CI)	OR	(95 % CI)	OR	(95 % CI)	OR	(95 % CI)
**Age group**								
11 years	1.00		1.00		1.00		1.00	
13 years	0.74	(0.63–0.87)	0.87	(0.74–1.03)	1.36	(1.09–1.70)	0.68	(0.58–0.80)
15 years	0.52	(0.45–0.61)	0.73	(0.62–0.87)	1.33	(1.06–1.65)	0.48	(0.41–0.57)
**Family affluence**								
Low	1.00		1.00		1.00		1.00	
Medium	1.16	(0.98–1.38)	1.14	(0.95–1.38)	0.72	(0.58–0.89)	1.16	(0.98–1.38)
High	1.76	(1.41–2.18)	1.60	(1.27–2.02)	0.54	(0.40–0.74)	1.76	(1.41–2.18)
**Sex**								
Boys	1.00		1.00		1.00		1.00	
Girls	1.57	(1.38–1.78)	1.59	(1.39–1.83)	0.61	(0.51–0.73)	0.73	(0.64–0.82)

OR = Odds ratio, CI = Confidence interval, WHO = World Health Organization

^*^ WHO recommendations = 60 minutes of at least moderately intense physical activity on all seven days of the week

^**^ Low levels of physical activity = 60 minutes of at least moderately intense physical activity on less than three days per week

**Table 3:** Daily intake of fruit and vegetables, non-daily consumption of soft drinks, as well as breakfast daily by sex, age and family affluence (n=2,280 girls, n=2,002 boys)^*^ Source: 2017/18 German HBSC study

	Fruit daily		Vegetables daily		Soft drinks not daily		Breakfast daily	
	%	(95 % CI)	%	(95 % CI)	%	(95 % CI)	%	(95 % CI)
**Girls**	**42.1**	**(30.0–44.2)**	**31.4**	**(29.4–33.4)**	**88.5**	**(87.1–89.8)**	**50.6**	**(48.5–52.8)**
**Age group**								
11 years	48.5	(44.6–52.4)	32.7	(29.1–36.4)	89.1	(86.4–91.4)	63.1	(59.2–66.8)
13 years	43.1	(39.4–47.0)	33.2	(29.7–36.9)	87.6	(84.8–89.9)	50.9	(47.0–54.7)
15 years	35.7	(32.5–39.1)	28.8	(25.7–32.0)	88.9	(86.4–90.9)	39.9	(36.5–43.3)
**Family affluence**								
Low	37.6	(33.0–42.5)	28.0	(23.8–32.6)	85.4	(81.5–88.6)	39.0	(34.3–43.9)
Medium	39.8	(37.1–42.5)	29.4	(27.0–31.9)	88.2	(86.3–89.9)	50.7	(48.0–53.5)
High	55.1	(50.0–60.2)	42.0	(37.0–47.2)	92.9	(89.8–95.1)	64.1	(59.0–68.9)
**Boys**	**32.1**	**(30.0–34.3)**	**22.7**	**(20.9–24.7)**	**82.5**	**(80.6–84.2)**	**59.0**	**(56.7–61.3)**
**Age group**								
11 years	41.4	(37.4–45.4)	28.5	(25.0–32.3)	87.0	(83.9–89.6)	65.5	(61.5–69.2)
13 years	31.4	(27.9–35.2)	21.3	(18.2–24.6)	80.6	(77.2–83.6)	58.1	(54.1–62.0)
15 years	23.5	(20.3–27.0)	18.5	(15.6–21.8)	80.0	(76.5–83.0)	53.4	(49.4–57.3)
**Family affluence**								
Low	28.3	(23.3–33.9)	19.5	(15.3–24.5)	77.2	(71.7–81.8)	46.2	(40.3–52.1)
Medium	31.9	(29.3–34.6)	22.7	(20.4–25.1)	83.5	(81.3–85.5)	60.2	(57.4–63.0)
High	37.7	(32.2–43.6)	24.2	(19.6–29.6)	85.0	(80.1–88.9)	68.2	(62.4–73.4)

CI = Confidence interval

^*^ Missing values lead to different numbers of respondents for individual indicators.
